# Defective Apoptosis in Intestinal and Mesenteric Adipose Tissue of Crohn’s Disease Patients

**DOI:** 10.1371/journal.pone.0098547

**Published:** 2014-06-02

**Authors:** Cilene Bicca Dias, Marciane Milanski, Mariana Portovedo, Vivian Horita, Maria de Lourdes Setsuko Ayrizono, Núria Planell, Cláudio Saddy Rodrigues Coy, Lício Augusto Velloso, Luciana Rodrigues Meirelles, Raquel Franco Leal

**Affiliations:** 1 Coloproctology Unit, Surgery Department, University of Campinas (UNICAMP), Medical School, Sao Paulo, Brazil; 2 Laboratory of Cell Signaling, Internal Medicine Department, University of Campinas (UNICAMP), Medical School, Sao Paulo, Brazil; 3 Doctoral CAPES fellowship, Post graduate Program in Surgery Sciences, Faculty of Medical School, University of Campinas, Sao Paulo, Brazil; 4 Department of Pathology, University of Campinas (UNICAMP), Medical School, Sao Paulo, Brazil; 5 Department of Gastroenterology and Bioinformatics Platform, CIBERehd, Barcelona, Spain; CWRU/UH Digestive Health Institute, United States of America

## Abstract

**Background:**

Crohn’s disease (CD) is associated with complex pathogenic pathways involving defects in apoptosis mechanisms. Recently, mesenteric adipose tissue (MAT) has been associated with CD ethiopathology, since adipose thickening is detected close to the affected intestinal area. However, the potential role of altered apoptosis in MAT of CD has not been addressed.

**Aims:**

To evaluate apoptosis in the intestinal mucosa and MAT of patients with CD.

**Methods:**

Samples of intestinal mucosa and MAT from patients with ileocecal CD and from non-inflammatory bowel diseases patients (controls) were studied. Apoptosis was assessed by TUNEL assay and correlated with the adipocytes histological morphometric analysis. The transcriptional and protein analysis of selected genes and proteins related to apoptosis were determined.

**Results:**

TUNEL assay showed fewer apoptotic cells in CD, when compared to the control groups, both in the intestinal mucosa and in MAT. In addition, the number of apoptotic cells (TUNEL) correlated significantly with the area and perimeter of the adipose cells in MAT. Transcriptomic and proteomic analysis reveal a significantly lower transcript and protein levels of Bax in the intestinal mucosa of CD, compared to the controls; low protein levels of Bax were found localized in the *lamina propria* and not in the epithelium of this tissue. Furthermore, higher level of Bcl-2 and low level of Caspase 3 were seen in the MAT of CD patients.

**Conclusion:**

The defective apoptosis in MAT may explain the singular morphological characteristics of this tissue in CD, which may be implicated in the pathophysiology of the disease.

## Introduction

The pathophysiology of CD is not yet completely elucidated, but environmental factors and inappropriate responses of the immune system in genetically-susceptible individuals have been proposed as possible causes of the disease. [1]–[3] A common feature in chronic CD with transmural inflammation is hypertrophy of the mesenteric adipose tissue (MAT), close to the affected intestinal area; furthermore, the potential involvement of this phenomenon in the disease’s pathophysiology has been recently suggested. This alteration extends from the mesentery, partially covers the circumference, presents an outer layer of intestinal fat and may involve the small and large bowel. [4] Differential expression of adipocytokines and pro-inflammatory cytokines, as well as, histological alterations have been previously described in the MAT of CD individuals. [5]–[8] However, no studies regarding apoptosis pathways in this tissue have been yet reported.

Apoptosis is a known physiological process of programmed cell death and is essential for the development and homeostasis of tissues and organs as well as the elimination of hazards and abnormal cells. [9], [10] In the past 30 years, due to the importance of this cellular mechanism in many diseases, methods have been developed for the detection of apoptosis and of the proteins involved in the process. Apoptosis can be induced by two main pathways: the intrinsic (mitochondrial), in which Bax is one of the most important pro-apoptotic protein, and the extrinsic pathways. [11] In addition, there is a close relationship between these apoptosis-related pathways and inflammatory pathways. TNF-α, an important pro-inflammatory cytokine, is involved in the activation of apoptosis, while NF-κB has an anti-apoptotic function, activating the expression of other members of the Bcl-2 family, such as Bcl-2, which prevents cell death [12], [13].

While apoptosis in MAT has not yet been investigated in CD, studies regarding apoptosis in the intestinal tissue of CD patients, and in other inflammatory bowel diseases, such as, ulcerative colitis (UC) and in the ileal pouch of UC patients, have been previously published. [14]–[16] Reports show that the T cells of CD mucosa exhibit resistance to a variety of signals that induce apoptosis, including the differential expression of proteins from the Bcl-2 family and differences in the ratio between pro and anti-apoptotic proteins [10], [17], suggesting that apoptosis may be one of the mechanisms involved in CD pathophysiology. Furthermore, defective apoptosis in immune cells, such as macrophages and neutrophils, has been reported [18], [19].

Whether the thickening of MAT acts as a barrier to the inflammatory process, or is a secondary factor that maintains the inflammatory process, resulting in the transmural aspect of CD, is unknown. [4], [20], [21] Therefore, this study aimed to evaluate the potential contribution of apoptosis in accumulation of MAT, as well as the relationship between altered apoptosis in MAT and in intestinal tissue involved by CD. To do this, we detected apoptotic DNA strand breaks using the TUNEL assay, in addition to analyzing the transcriptional and protein expressions of selected molecules, to determine the pathways potentially involved in altered apoptosis.

## Materials and Methods

### Sample Collection

Intestinal mucosal and MAT samples, located near the affected intestinal area, were taken from 10 patients with ileocecal CD who underwent surgical resection [median age, 34.9 (range, 14–60) years; 50% male]. We labeled as ICD group for intestinal mucosa of CD patients and ACD for MAT of these patients. The presence of disease activity was assessed by colonoscopy before surgery and all patients had a Crohn’s disease activity index (CDAI) [22] of more than 250 points. The control groups were composed of 8 patients who underwent intestinal resection for non-inflammatory disease, with normal distal ileum (MAT control group – AC group) [median age, 55.6 (range, 39–70) years; 62.5% male; 37.5% female], and 8 patients with normal ileocolonoscopy (control intestinal tissue group – IC group) [median age, 50.4 (range, 33–60) years; 37.5% male; 62.5% female]. All CD patients and healthy controls had body mass index, (weight in kilograms (kg) divided by height in meters squared) (BMI) less than 25 points.

### TUNEL Apoptosis Detection Analysis

For detection and quantification of apoptosis in the intestinal mucosa and MAT samples, the TUNEL Apoptosis Detection assay was performed (Terminal deoxynucleotidyl transferase mediated dUTP nick end labelling) [23], using a kit from Millipore (Billerica, MA). This assay is based on the marking of DNA strand breaks by the technique of labelling of DNA with terminal dUTP (FITC-conjugated). We used the protocol recommended by the manufacturer. The nuclear staining was performed with propidium iodide (PI). Photomicrographs were taken using a Leica DM 4500B microscope and Leica DFC 290 digital camera system with Leica Application Suite version 3.8 Software (Leica Microsystems, Wetzlar). Three fields for each sample were captured. Any cell type showing nuclear co-labeling (FITC+PI) was considered positive for quantitative analysis, which was analyzed by a blinded observer (C.B.D.), in a panchromatic objective field of higher magnification 40X.

### Histological Analysis (Hematoxylin - Eosin)

Biopsies from the mucosa of the terminal ileum and from the MAT, near the affected intestinal area, were embedded in paraffin blocks for histological analysis. Sections of 5 µm were cut and stained with hematoxylin and eosin dye.

Photomicrographs were taken using a Zeiss Axiophot microscope and Cannon Power Shot G5 digital camera system (Cannon Inc., Tokyo). Fifty fields of higher magnification (40X) were scanned for each sample and 10 random fields were analyzed. The number of adipocytes was counted and their area and perimeters were obtained. The morphometric results were quantified by a blinded observer (C.B.D.) using the software Image J (Image Processing and Analysis in Java, public domains, *rsbweb.nih.gov/ij/21*).

### Bax, Bcl-2 and Ki67 Immunohistochemical Staining

Histological sections of 5 µm were also performed for immunostaining procedures of samples included in paraffin blocks. Endogenous peroxidase was blocked with 3% hydrogen peroxide/10 mM PBS pH 6.0 for 15 min. Afterwards, the sections were microwaved in 3% milk buffer for 30 min and incubated overnight with primary antibodies; anti-Bax (DAKO A/S Denmark; N-20 5c493, rabbit polyclonal), anti-Bcl-2 (DAKO A/S Denmark; N-19 5c492, mouse polyclonal), anti-Ki67 (DAKO A/S Denmark; F0788, mouse monoclonal) with dilutions of 1∶600, 1∶150 and 1∶500 respectively at 20°C. The sections were incubated with post primary block and secondary antibodies (Novocastra Laboratories Ltd; Novolink RE 7260-K) for 1 h, and processed using the DAB reaction (0.5 mg/ml, Sigma, USA, St Louis). Any cell type showing cytoplasmic staining was considered positive for quantitative analysis [11], [24], which was performed by a blinded observer (C.B.D.). The microscope and the software used to capture images for quantitative analysis were the same as those used for the hematoxylin and eosin study.

### Caspase 3 Immunofluorescence Staining

Histological sections of 5 µm were also performed for immunofluorescence procedures of samples included in paraffin blocks. The preparation of slides was performed (deparaffinization and hydration), followed by antigen retrieval. The tissue was incubated in primary antibody anti-Caspase 3 (Santa Cruz CA; H-277: sc-7148, rabbit polyclonal), with a dilution of 1∶200 at 4°C overnight and after with secondary antibody conjugated with FITC (goat anti-rabbit IgG-FITC: sc-2012) in the same concentration for 1 hour. DAPI was used for nuclear staining. Any cell type showing co-labeling in the cytosol for FITC were considered positive for quantitative analysis [11], which was performed by a blinded observer (C.B.D.). The microscope and the software used to capture images for quantitative analysis were the same as those used for the TUNEL study.

### RT-PCR Analysis

Biopsies from the mucosa of the terminal ileum and from the MAT, located near the affected intestinal area, were snap-frozen in liquid nitrogen and stored at −80°C until use. Total RNA was extracted using Trizol (Invitrogen), according to the manufacturer’s instructions. RNA purity and concentration were determined by UV spectrophotometry at 260 nm. RNA was treated with RNase-free Dnase (RQ1 RNase-free Dnase, Promega) and then reverse transcribed using oligo (dT) primers and reverse transcriptase (RevertAid Kit, Fermentas). The reaction mixture (20 µl) was incubated at 42°C for 60 min, then for 10 min at 70°C, and cooled on ice. RT-PCR was performed on resulting cDNA, using the manufacturer’s protocol, in a 25 µl reaction volume per capillary. Gene-specific primers (Applied Biosystems) were: Hs00180269_m1 (Bax); Hs00608023_m1 (Bcl2); NM_002046.3 (GAPDH). RT-PCR amplification consisted of an initial denaturation step (50°C for 2 min and 95°C for 10 min), 40 cycles of denaturation (95°C for 15 s), annealing (53°C for 20 s) and extension (72°C for 20 s), followed by a final incubation at 60°C for 1 min. All measurements were normalized by the expression of GAPDH gene, considered as a stable housekeeping gene. Gene expression was determined using the delta-delta Ct method: 2^−ΔΔCT^ (ΔΔCT = [Ct(target gene)–Ct(GAPDH)]_patient_–[Ct(target gene)–Ct(GAPDH)]_control_).

Real-time PCR analysis of gene expression was performed in a 7500 SDS sequence detection system (Applied Biosystems). The optimal concentration of cDNA and primers, as well as the maximum efficiency of amplification, were obtained by five-point, two-fold dilution curve analysis for each gene. Real-time data were analyzed using the Sequence Detector System 1.7 (Applied Biosystems).

### Immunoblotting – Gel Electrophoresis

For total protein extract preparation, the fragments of MAT, which were previously snap-frozen and stored at −80°C, were homogenized in solubilization buffer at 4°C [1% Triton X-100, 100 mM Tris-HCl (pH 7.4), 100 mM sodium pyrophosphate, 100 mM sodium fluoride, 10 mM EDTA, 10 mM sodium orthovanadate, 2.0 mM phenylmethylsulfonyl fluoride (PMSF), and 0.1 mg aprotinin/ml] with a Polytron PTA 20S generator (model PT 10/35; Brinkmann Instruments, Westbury, NY) operated at maximum speed for 30 sec. Insoluble material was removed by centrifugation (20 min at 11000 rpm at 4°C). The protein concentrations of the supernatants were determined by the Bradford dye binding method. [25] Aliquots of the resulting supernatants containing 50 µg total proteins were separated by SDS-PAGE, transferred to nitrocellulose membranes and blotted with anti-Bax and anti-Bcl-2 antibodies [26].

Reagents for SDS-PAGE and immunoblotting were from Bio-Rad Laboratories (Richmond, CA). Phenylmethylsulfonyl fluoride, aprotinin, Triton X-100, Tween 20, and glycerol were from Sigma (St. Louis, MO). Nitrocellulose paper (BA85, 0.2 µm) was from Amersham (Aylesbury, UK). The anti-Bax (sc-493, rabbit polyclonal) and anti-Bcl-2 (sc-492, rabbit polyclonal) antibodies were from Santa Cruz Biotechnology, Inc. (Santa Cruz, CA). Molecular weights of proteins were assessed using the PageRulerTM from Fermentas (Glenburnie, MD). The signal was detected by a chemiluminescent reaction (SuperSignal West Pico Chemiluminescent Substrate from Pierce Biothecnology, Inc. Rockford, IL).

The results of blots are presented as direct comparisons of bands in autoradiographs and were quantified by densitometry using the Gel-Pro Analyzer 6.0 software (Exon-Intron Inc., Farrell, MD). All results were normalized by β-actin.

### Statistical Analysis

The Pearson’s regression coefficient (r) was used for correlations between TUNEL results and morphometric data. A non-parametric test (Mann-Whitney U, unpaired) was performed using R Statistics Software (version 2.15.0) for statistical analyses to compare the MAT of the CD group and its respective adipose control group. The intestinal tissue of the CD group and its respective intestinal control group were also compared separately. The level of significance was set at p<0.05.

### Ethical Considerations

The study was performed in accordance with the Declaration of Helsinki and was approved by the Institutional Ethics Committee of the Clinical Hospital of the Faculty of Medical Sciences (University of Campinas, Sao Paulo, Brazil). All biopsies were obtained after patients gave their written informed consent. In the case of minors/teenagers enrolled in our study, parents or guardians signed the informed consent on their behalf. All consents forms are kept as hard copy. The study was carried out in the Coloproctology Unit of the Surgery Department, and at the Cell Signaling Laboratory of the Department of Internal Medicine, University of Campinas.

## Results

### TUNEL Assay Reveals Altered Apoptosis in the Intestinal Mucosa and in MAT of CD Individuals

**Figure 1:**
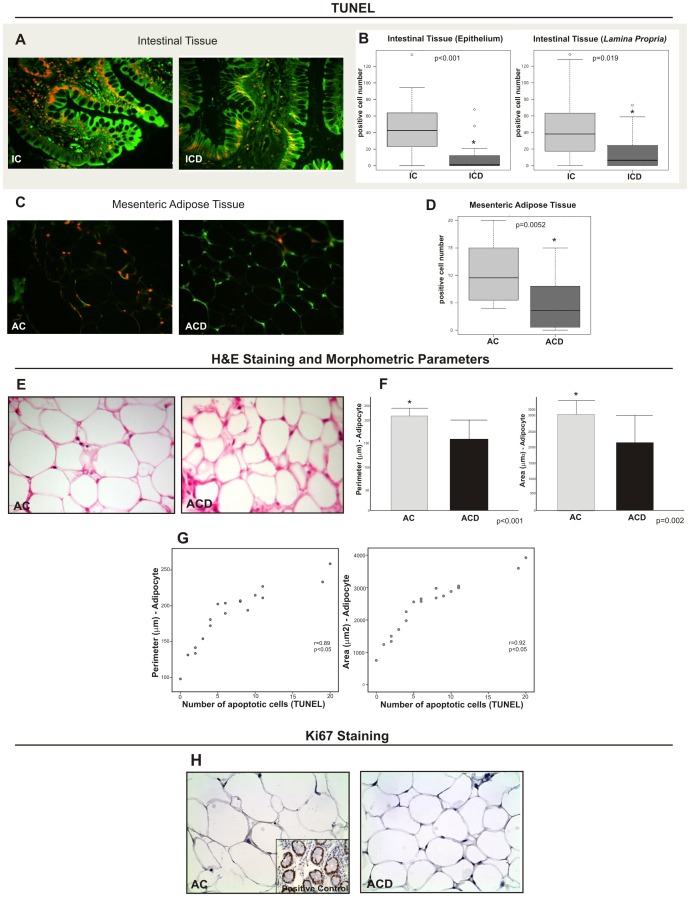
TUNEL assay shows different patterns in the intestinal mucosa (ICD) and in the mesenteric adipose tissue (MAT) of Crohn’s disease (ACD), compared to the respective control biopsy samples (IC and AC). (A) Enterocyte and *lamina propria* cell apoptosis are shown by immunofluorescence staining (overlay image); TUNEL+ cells are showed in orange (co-labeled by PI and FITC). Low numbers of TUNEL+ enterocytes and *lamina propria* cells were detected in the ICD group compared to IC. (C) Adipocyte apoptosis, shown by immunofluorescence staining (overlay image); TUNEL+ cells are showed in orange (co-labeled by PI and FITC). Low numbers of TUNEL+ adipocytes were detected in the ACD group compared to AC. Note the high density of TUNEL+ adipocytes, in orange, in the control (AC). Images were obtained using a 40x objective. (B) and (D) Quantitative analysis of TUNEL staining in the ICD and ACD groups, compared to the respective controls (IC and AC). The graphs of intestinal tissue show the quantitative analysis for the epithelium and *lamina propria* TUNEL staining, separately. For ICD, n = 10; for ACD, n = 10; for IC, n = 8; and, for AC, n = 8, *p<0.05 vs control. (E) Representative hematoxylin-eosin (H&E) staining of fixed paraffin-embedded MAT from AC and ACD groups shows lower area and perimeter of the adipocytes in the ACD group, compared to the control (AC). Images were obtained using a 40x objective. (F) Quantitative morphometric histological analysis in the mesenteric adipose tissue (MAT) of Crohn’s disease (ACD), compared to the respective control group (AC). The graphs show the decreased perimeter (µm) and area (µm^2^) of the adipocytes from the MAT of Crohn’s disease, compared to the control biopsy samples. For ACD, n = 10; for AC, n = 8, *p<0.05 vs control. (G) The graphs dispersion show a significant correlation between the perimeter (µm) and the number of apoptotic cells (TUNEL+), (r = 0.89, p<0.05) and also between the area (µm^2^) and the number of apoptotic cells (TUNEL+), (r = 0.92, p<0.05). (H) Immunohistochemical staining of Ki67 in the mesenteric adipose tissue (MAT) of the Crohn’s disease group (ACD), compared to the control biopsy samples (AC); no evidence of proliferation were found in all samples (ACD, n = 10; AC, n = 8). Images were obtained using a 40x objective. The positive control was from tissue section of intestinal mucosa.

The macroscopic increase of MAT close to the affected intestinal area is a common feature in CD, in contrast to observations in other inflammatory bowel diseases. Therefore, our first objective was to determine, using TUNEL assay, the overall rate of apoptotic cells in intestinal mucosa and MAT samples of CD, and compare with their respective controls. This analysis showed a significantly lower number of apoptotic cells in the ICD and ACD groups, when compared to the respective control groups (IC and AC) (p<0.05). A representative image of TUNEL assay is shown in Figure 1 (A and C), where the apoptotic cells are well identified in orange in the different groups. Figure 1 (B and D) shows the quantitative analysis for all assessed samples.

Since we found an impaired apoptosis in the MAT of CD group compared to controls, one of our aims was also to analyze the morphometric characteristics of MAT, near the intestinal affected area, and correlate these findings with the apoptotic index (TUNEL). Figure 1 (E and F) demonstrates the histological aspect (hematoxilin and eosin) and the mean perimeter and area of the adipose cells of the AC and ACD groups. The adipocytes of the ACD group presented a lower mean area and perimeter, when compared to the controls (AC group) (p<0.05). There was a strong positive correlation between the adipocyte area and perimeter with the apoptotic index, as shown in Figure 1G. In addition, immunohystochemistry for Ki67 was performed in all samples from ACD and AC groups to access the proliferation rate of the adipocytes and verify if this could be related to the morphometric characteristics of MAT. However, no evidence of proliferation was verified in MAT from both groups (Figure 1H).

### Transcriptional Expression of Apoptosis-related Genes in the Intestinal Mucosa and in MAT Reveals differences between CD and Controls

**Figure 2:**
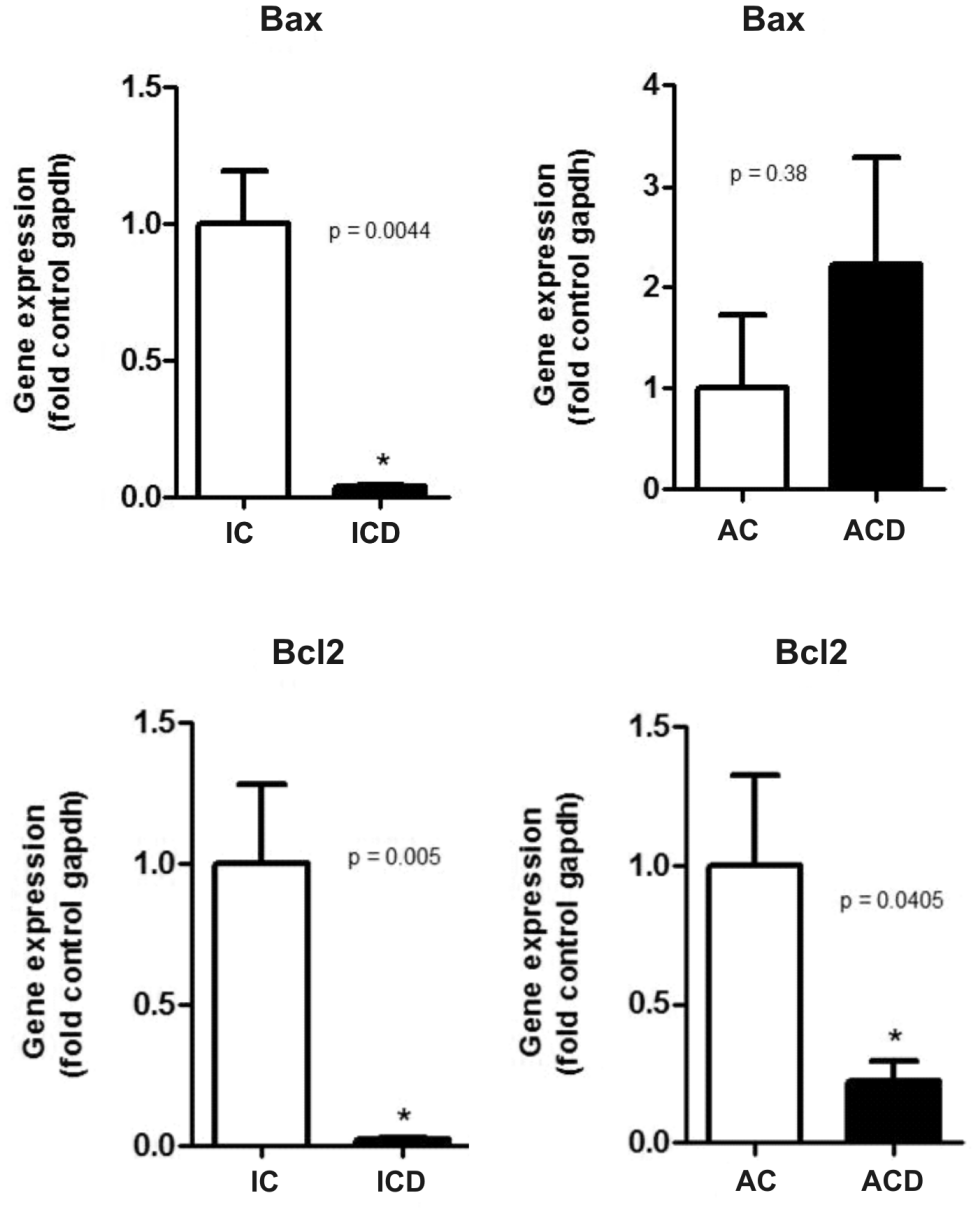
Bax and Bcl2 gene expressions, as determined by RT-PCR; low transcript levels of Bax and Bcl2 are observed in the intestinal mucosa of the Crohn’s disease group (ICD), compared to the respective control (IC). Low transcript levels of Bcl2 are also seen in the mesenteric adipose tissue (MAT) of the Crohn’s disease group (ACD), compared to the respective control (AC), while no differences in Bax transcripts were found in the MAT groups. For ICD, n = 10; for ACD, n = 10; for IC, n = 8; and, for AC, n = 8, *p<0.05 vs control.

Since alterations were detected in overall rate of apoptosis by TUNEL in intestinal mucosa and in MAT of CD patients compared to the controls, we decide to explore the molecular mechanisms involved. Two relevant genes related to apoptosis pathway were studied; Bax and Bcl2, which encode for a pro-apoptotic and an anti-apoptotic protein, respectively. We found a significant decrease of Bax transcriptional expression in the intestinal mucosa of patients with CD, compared to the control (p<0.05), while no differences were detected in MAT (p>0.05). In addition, Bcl2 transcriptional expression was significantly decreased in the intestinal mucosa and in MAT in CD, when compared to the respective controls (p<0.05). These findings are shown in Figure 2.

### Protein Analysis by Immunohistochemistry Confirms Transcriptional Expression of Bax in the Intestinal Mucosa of CD Patients

**Figure 3:**
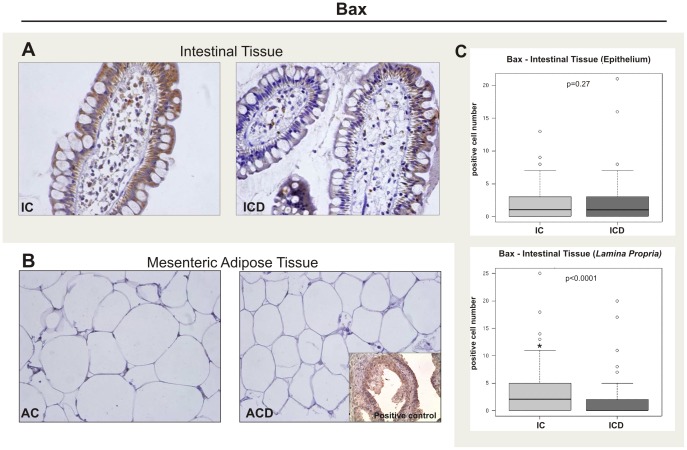
Immunohistochemical staining of Bax in the intestinal tissue (epithelium and *lamina propria*) of the Crohn’s disease group (ICD) and in the mesenteric adipose tissue (MAT) of the Crohn’s disease group (ACD), compared to the respective control biopsy samples (IC and AC). (A) Representative staining of fixed paraffin-embedded tissue of terminal ileum from IC and ICD groups showing fewer positive cells (brown) in the *lamina propria* of ICD, compared to the IC group. (B) Representative staining of fixed paraffin-embedded mesenteric adipose tissue in the AC and ACD groups; no differences were found among the groups. Images were obtained using a 40x objective. The positive control was from tissue section of prostatic cancer. For ACD, n = 10; and for AC, n = 8. (C) Quantitative analysis of immunohistochemical staining for Bax in the intestinal mucosa of the Crohn’s disease group (ICD), compared to the respective control (IC). The graphs show the quantitative analysis for the epithelium and *lamina propria* immunostainings separately. For ICD, n = 10; for IC, n = 8, *p<0.05 vs control.

In order to validate our transcriptional finding we used immunohistochemistry in the same samples used in PCR analysis. Figure 3 (A and B) shows a representative picture of Bax staining for intestinal tissue and MAT. A clear positive immunoreactivity was observed for intestinal tissue, while MAT samples were all negative. The quantitative analysis reveals a significant decrease in Bax expression restricted in the *lamina propria* of CD patients, clearly shown in Figure 3 (A and C), according with transcriptional results. However, no differences were found in epithelium from intestinal mucosa.

### Immunohistochemical Protein Analysis Revealed a Higher Expression of Bcl-2 in MAT of CD Patients

**Figure 4:**
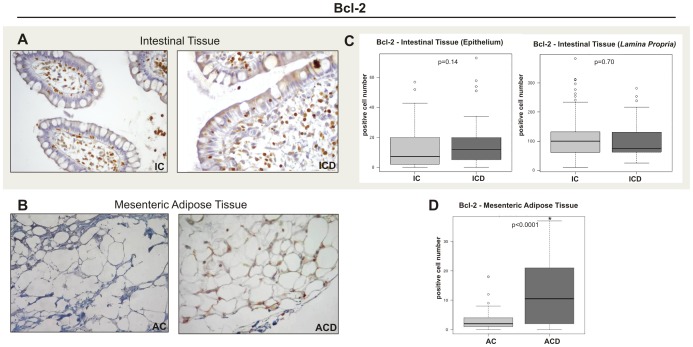
Immunohistochemical staining of Bcl-2 in the intestinal tissue (epithelium and *lamina propria*) of the Crohn’s disease group (ICD) and in the mesenteric adipose tissue (MAT) of the Crohn’s disease group (ACD), compared to the respective control biopsy samples (IC and AC). (A) Representative staining of fixed paraffin-embedded tissue of terminal ileum from IC and ICD groups showing similar numbers of positive cells (brown) in the ICD and IC groups. (B) Representative staining of fixed paraffin-embedded mesenteric adipose tissue from the AC and ACD groups, showing a higher intensity in the ACD group, compared to the control (AC). Images were obtained using a 40x objective. (C) and (D) Quantitative analysis of immunohistochemical staining for Bcl-2 of ICD and ACD groups, compared to the respective control groups (IC and AC). The graphs of intestinal tissue show the quantitative analysis for the epithelium and *lamina propria* immunostainings separately. For ICD, n = 10; for ACD, n = 10; for IC, n = 8; and for AC, n = 8, *p<0.05 vs control.

CD patients presented significantly higher protein expression of Bcl-2 in MAT, compared to the controls, as seen in Figure 4 (B and D). Positive immunoreactivity for Bcl-2 was also observed in the intestinal mucosa (Figure 4A); however, quantitative analysis showed no statistical difference between the groups (p>0.05) (Figure 4C).

### Confirmation of the Expression of Bax and Bcl-2 Proteins in the Intestinal Mucosa and in MAT of CD Patients by Immunoblotting

**Figure 5:**
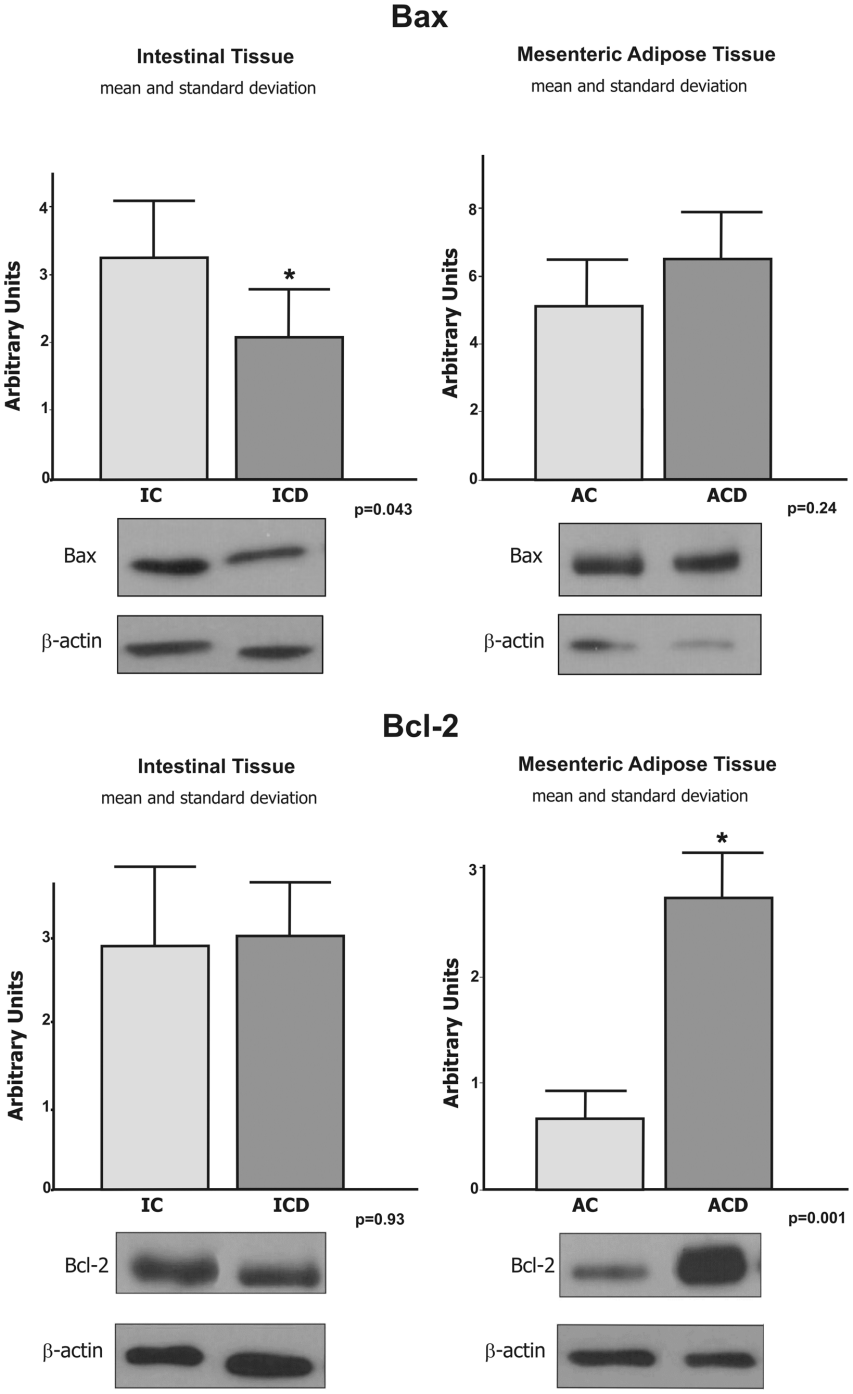
Representative Western blot analyses and determination of Bax and Bcl-2 protein expression in the intestinal tissue (mucosa) of the Crohn’s disease group (ICD) and in the mesenteric adipose tissue (MAT) of the Crohn’s disease group (ACD), compared to the respective controls (IC and AC). Decreased expression of Bax was observed in ICD group, compared to the control group (IC), and higher expression of Bcl-2, an anti-apoptotic protein, was observed in the MAT of Crohn’s disease (ACD) compared to the control group (AC). For illustration purposes, each band represents one patient. For ICD, n = 10; for ACD, n = 10; for IC, n = 8; and, for AC, n = 8, *p<0.05 vs control.

Due to the conflicting data for Bcl2 transcriptional expression and the immunohistochemistry study of Bcl-2, we assessed protein expression by immunoblotting. The Bcl-2 anti-apoptotic protein was found to be significantly more expressed in the MAT of CD patients (ACD group), when compared to controls (AC group) (p<0.05). No differences were detected in the intestinal mucosa between the ICD and IC groups (p>0.05). See Figure 5.

Immunoblotting for Bax, shown in Figure 5, was also performed in intestinal mucosa and MAT from CD and controls. The levels of Bax were in accordance to the transcriptional results.

### Caspase 3 Expression Confirms Defective Apoptosis in MAT of CD Patients

**Figure 6:**
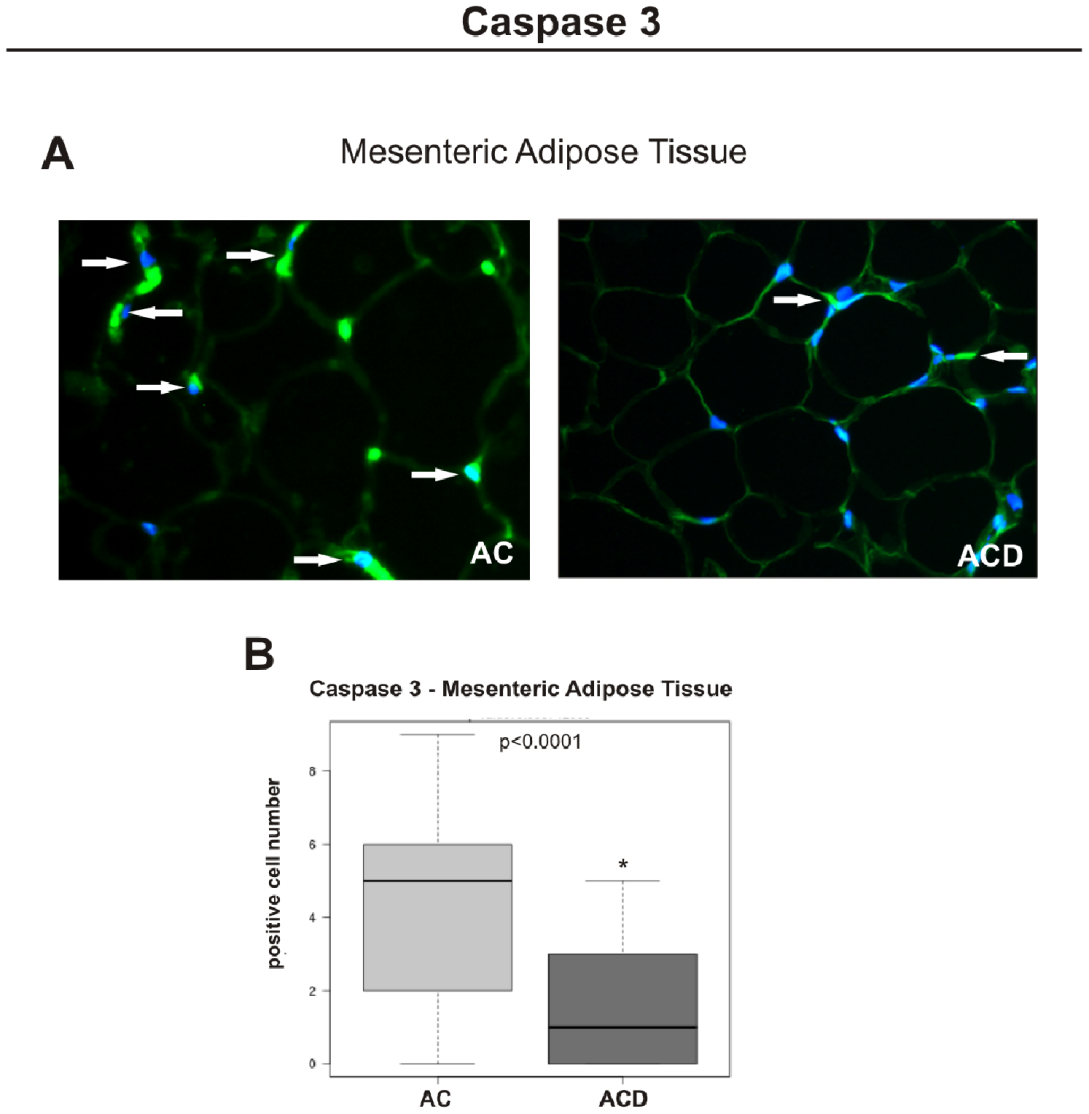
Immunofluorescence staining of Caspase 3 in the mesenteric adipose tissue (MAT) of the Crohn’s disease group (ACD) compared to the respective control biopsy samples (AC). (A) Representative staining of fixed paraffin-embedded mesenteric adipose tissue from the AC and ACD groups, showing a higher number of positive cells for FITC (green-fluorescent) in the cytosol, co-labeled with DAPI (nuclear staining: blue-fluorescent) in the ACD group, compared to the control (AC). The arrows show the positive cells. Images were obtained using a 40x objective. (B) Quantitative analysis of immunofluorescence staining for Caspase 3 of ACD group compared to the respective control group (AC). For ACD, n = 10; for AC, n = 8, *p<0.05 vs control.

To confirm the TUNEL and protein-related apoptosis expression results, immunofluorescence for Caspase 3 was performed. The Caspase 3 was significantly less expressed in the MAT of CD patients (ACD group), when compared to controls (AC group) (p<0.05). Figure 6 illustrates this finding.

## Discussion

Although phenotypic variation occurs in CD patients, some common macroscopic aspects can be observed, especially with regard to the thickening of the MAT close to the affected intestinal area. This feature is not seen in patients with UC who develop a superficial inflammatory process in the intestinal wall that is usually restricted to the intestinal mucosa and submucosa layers. [7], [27] The adipose tissue is considered an important endocrine organ, responsible for the production and release of hormones and cytokines. [28] It is known that mesenteric adipocytes of normal individuals are able to synthesize several pro-inflammatory and anti-inflammatory cytokines, and express Toll-Like Receptor 4 for the recognition of bacterial antigens. [29] These studies revealed abnormalities in the MAT of CD patients, including the infiltration of macrophages and T cells, perivascular inflammation, fibrosis and differences in adipocytes number and size.

There are currently no studies regarding apoptosis in the MAT of CD individuals, nor in the animal model of hypertrophied MAT with associated colitis. [30] With this purpose in mind, we used TUNEL assay to evaluate apoptosis in the intestinal mucosa and in MAT, which revealed significantly fewer apoptotic cells in CD, when compared to the control groups, not only in the intestinal mucosa, but also in MAT. Intestinal barrier is maintained due to balance rates of epithelial cell proliferation and cell death. The literature has shown that healthy intestinal mucosa has high rates of cell proliferation at the base of the epithelium (crypt), with inhibition of signals to apoptosis, whereas epithelial cells that compound the intestinal villi shows cell death activation. This mechanism is not totally understood, but it seems to be a shedding cell associated to cell death. [31]–[33] This explains the high turn-over of the intestinal epithelial cells in homeostasis conditions. In the present study, we found a low amount of TUNEL-positive epithelial cells in CD compared to controls. This may be explained by the presence of damaged mucosa consequent to inflammation, where most part of the villi are lost. In this situation, the remaining cells may be the ones that show low rate of apoptosis and high rate of proliferation in order to recover the affected area and restore function. Concerning the decreased apoptosis in *lamina propria* cells of CD compared to the controls, and the fact that part of these cells are immune cells, this confirms previous published results in the literature [10], [17].

In addition, the main novelty of our study was the correlation of the number of apoptotic cells in MAT, as evidenced by TUNEL, with reduced adipocyte size. Peyrin-Biroulet et al. [8] previously described the morphometric features of the adipocytes from hypertrophied CD MAT relating that these cells were small in size and four times higher in number when compared to control adipose tissue. However, these authors did not correlate these findings with apoptosis. Figure 1G shows the significant correlation between the morphometric parameters (perimeter and area) of the adipose cells and the number of the apoptotic cells in the MAT of CD and controls. These findings may explain, at least in part, the intriguing features of MAT in CD: the thickening of this tissue may be due to a resistance of adipose cells to undergo apoptosis, leading to an increased number of adipocytes that exhibit a lower perimeter and area than the control group. NF-KB activation is one of the mechanisms described that can inhibit apoptosis by inducing Bcl-2 expression (anti-apoptotic protein). [34], [35] High levels of NF-KB activation are verified in CD [36], [37]. This factor is responsible for activate transcription of a large number of genes related to inflammation, among them, TNF-α transcription. This may explain the resistance to apoptosis in MAT of CD patients. We did not verify positivity for Ki67 in MAT from CD patients and controls. This result is in accordance with what is described in the literature concerning fat cell turn over in humans. In non-obese conditions, adipose cells are not prone to proliferation. Adipocytes proliferation (hyperplasia) occurs only in severe cases of obesity, while hypertrophy occurs across all obese states. [38] All CD patients included in the present study, as well as the healthy controls were not obese, presenting BMI (body mass index) less than 25.

To describe the molecular mechanisms involved with the defective apoptosis detected by TUNEL, we studied Bax and Bcl2 transcripts and also the respective encoded proteins in the intestinal mucosa and MAT of CD patients. Itoh et al. found low levels of Bax in CD *lamina propria* T cells, using flow cytometric analysis, when compared to UC and controls, indicating a resistance to apoptosis in CD. [10] In the present study, we report findings of low transcript and protein levels of Bax in the intestinal mucosa of CD, compared to the control intestinal mucosa. Moreover, we observed that the low protein levels of Bax (as seen by immunohistochemistry) were localized in the *lamina propria,* and not in the epithelium. Although there was low transcriptional expression of Bcl2 in the ICD group, no differences were observed with regard to Bcl-2 protein expression, as analyzed by immunohistochemistry in this group. These findings reinforce the data of Itoh et al. [10] and Santaolalla et al. [17], who associated the defective apoptosis in the *lamina propria* to the Bax-related pathways.

A defective apoptosis was also seen in the MAT of CD. This apoptosis correlated significantly with high levels of Bcl-2 and Caspase 3, and not with Bax protein expression. The low Bcl2 transcriptional expression observed in association with higher protein MAT levels of Bcl-2 (as detected by immunohistochemistry and immunoblotting) in the ACD group could be explained by cytosine methylation, which greatly increases the stability of the Bcl2 promoter (as described by Lin et al. [39]). Another possibility is that high Bcl-2 protein levels could induce a negative feedback control of Bcl2 gene transcription. The decreased expression of Caspase 3 in MAT of ACD group compared to the control (AC group) confirmed an altered apoptosis in this tissue.

The point of our study is to present new data that may help to explain the singular characteristics of MAT in CD patients. Given the current emphasis that has been given to the role of adipose tissue in gut homeostasis and inflammation [40], the defective apoptosis of MAT in CD may explain the high survival rate of these cells, which in large amount may express higher levels of pro-inflammatory mediators. For instance, significantly higher expression of C-reactive protein (CRP), an inflammatory marker, was detected in the MAT of CD compared to UC and controls. [41] Moreover, a comparison of adipocyte gene expression from MAT of CD and in healthy individuals showed up-regulation of pro-inflammatory genes and decrease of genes involving lipid metabolism [21].

MAT may have an important role in the maintenance of inflammation in CD, since the altered balance between pro-inflammatory and anti-inflammatory factors in this tissue, as well as defective autophagy, have been previously reported in the literature. [5], [42]–[48] Among these studies, one of them verified lower levels of adiponectin (anti-inflammatory properties) in peripheral serum and in MAT of active CD patients, revealing deficient anti-inflammatory conditions. [43] Moreover, this tissue may be involved in the maintenance of inflammation in the late stages of the disease, and in the mechanism that leads to relapses during the course of the disease. Therefore, the decreased apoptosis revealed in the present study, associated with already published previous data that have shown the capacity of the adipose cells to produce cytokines and its plasticity [42], [44], [49], could lead to insights for further research that may explain the complete role of MAT in CD.
